# STERILE APETALA modulates the stability of a repressor protein complex to control organ size in *Arabidopsis thaliana*

**DOI:** 10.1371/journal.pgen.1007218

**Published:** 2018-02-05

**Authors:** Na Li, Zupei Liu, Zhibiao Wang, Licong Ru, Nathalie Gonzalez, Alexandra Baekelandt, Laurens Pauwels, Alain Goossens, Ran Xu, Zhengge Zhu, Dirk Inzé, Yunhai Li

**Affiliations:** 1 State Key Laboratory of Plant Cell and Chromosome Engineering, CAS Center for Excellence in Molecular Plant Sciences, Institute of Genetics and Developmental Biology, Chinese Academy of Sciences, Beijing, China; 2 University of Chinese Academy of Sciences, Beijing, China; 3 College of Life Science, Hebei Normal University, Shijiazhuang, Hebei, China; 4 VIB Center for Plant Systems Biology, Technologiepark 927, Ghent, Belgium; 5 Department of Plant Biotechnology and Bioinformatics, Ghent University, Technologiepark 927, Ghent, Belgium; Peking University, CHINA

## Abstract

Organ size control is of particular importance for developmental biology and agriculture, but the mechanisms underlying organ size regulation remain elusive in plants. Meristemoids, which possess stem cell-like properties, have been recognized to play important roles in leaf growth. We have recently reported that the *Arabidopsis* F-box protein STERILE APETALA (SAP)/SUPPRESSOR OF DA1 (SOD3) promotes meristemoid proliferation and regulates organ size by influencing the stability of the transcriptional regulators PEAPODs (PPDs). Here we demonstrate that KIX8 and KIX9, which function as adaptors for the corepressor TOPLESS and PPD, are novel substrates of SAP. SAP interacts with KIX8/9 and modulates their protein stability. Further results show that SAP acts in a common pathway with KIX8/9 and PPD to control organ growth by regulating meristemoid cell proliferation. Thus, these findings reveal a molecular mechanism by which SAP targets the KIX-PPD repressor complex for degradation to regulate meristemoid cell proliferation and organ size.

## Author summary

Organ size is coordinately regulated by cell proliferation and cell expansion; however, the mechanisms of organ size control are still poorly understood. We have previously demonstrated that the *Arabidopsis* F-box protein STERILE APETALA (SAP)/SUPPRESSOR OF DA1 (SOD3) controls organ size by promoting meristemoid proliferation. SAP functions as part of a SKP1/Cullin/F-box (SCF) E3 ubiquitin ligase complex and modulates the stability of the transcriptional regulators PEAPODs (PPDs) to control organ growth. Here we show that KIX8 and KIX9 are novel substrates of SAP. KIX8 and KIX9 have been shown to form a transcriptional repressor complex with PPD and TOPLESS (TPL) to regulate leaf growth. We found that SAP interacts with KIX8/9 *in vitro* and *in vivo*, and modulates their protein stability. Further analyses indicate that SAP acts in a common pathway with KIX8/9 and PPD to control meristemoid proliferation and organ growth. These findings reveal that SAP regulates organ size by targeting the KIX-PPD repressor complex for degradation.

## Introduction

How plants control final organ size is an intriguing question in developmental biology. Organ size is also important for plant yield and biomass. Previous studies suggest that the developing organs possess intrinsic signals to control their final size, although plant growth is affected by various environmental factors[1–5]. However, how plants determine their organ size is still unclear.

Cell proliferation and cell expansion play a predominant role in determining plant organ growth. Leaf development in *Arabidopsis* provides a good model system for analyzing the coordination of these two important processes[6, 7]. After the leaf primordium is initiated, cells in the primordium divide continuously to generate new cells with small size. In the tip region of the leaf, cell division gradually ceases and cells begin to differentiate and expand. Then this cell differentiation domain spreads down, forming a cell-cycle arrest front that moves toward the leaf base[8, 9]. While most cells behind this cell-cycle arrest front exit cell division, the meristemoid cells that possess stem cell-like properties divide a few rounds and then form stomata or epidermal pavement cells [10, 11]. This proliferation of meristemoid cells is specific for dicot plants [12]. In *Arabidopsis*, meristemoid cells generate about 48% of all pavement cells in leaves[13], indicating that the amplifying division of these meristemoid cells contributes significantly to leaf size.

Several key factors have been revealed to influence leaf size by regulating cell division rate[14–16], the duration of cell division[17–29], or cell expansion[30–37]. However, how plants determine organ growth through meristemoid cell proliferation is largely unknown. *PEAPOD1* (*PPD1*) and *PPD2* were the first two genes identified to regulate leaf size by limiting meristemoid cell proliferation[8]. The tandemly repeated *PPD1* and *PPD2* genes encode two plant specific transcriptional regulators. Knock-out or down-regulation of *PPD* genes results in large and dome-shape leaves due to the prolonged proliferation of meristemoids[8, 12]. A recent study shows that PPD proteins interact with KIX8 and KIX9, which act as adaptors to recruit the transcription repressor TOPLESS (TPL)[12]. Thus, PPD, KIX and TPL may function as a repressor complex to control meristemoid proliferation and leaf growth[12].

We have recently reported that the F-box protein STERILE APETALA (SAP)/SUPPRESSOR OF DA1 (SOD3) positively regulates leaf growth by promoting meristemoid cell proliferation[38]. SAP promotes organ growth by targeting PPD proteins for degradation. The *ppd* mutant partially suppresses the organ growth phenotypes of *sod3-1*, suggesting that SAP may also target other proteins for degradation to control organ growth. Here we report that KIX8 and KIX9 are two novel targets of SAP. SAP interacts with KIX8 and KIX9 and modulates their protein stability. We further demonstrate that SAP acts with KIX and PPD in a common genetic pathway to control meristemoid cell proliferation and organ growth. These results reveal a novel genetic and molecular mechanism in which SAP targets the KIX-PPD repressor complex for degradation to control organ growth.

## Results

### SAP interacts with KIX8 and KIX9 *in vitro* and *in vivo*

**Fig 1:**
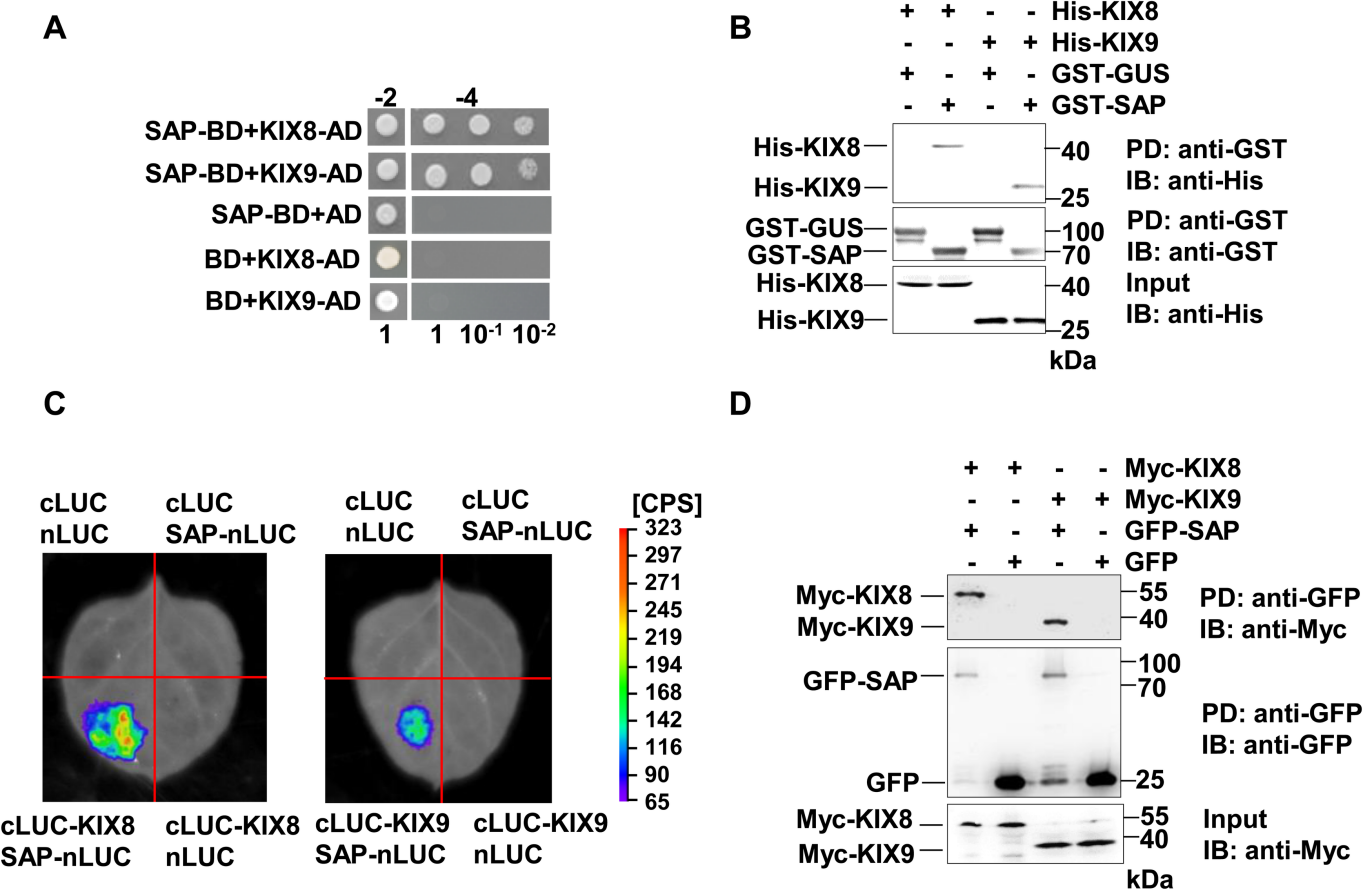
SAP interacts with KIX8 and KIX9. **(A)** SAP interacts with full length KIX8 and KIX9 in yeast cells. Transformants were selected on media -2 (SD/-Leu/-Trp), and interactions were tested on media -4 (SD/-Ade/-His/-Leu/-Trp) using a serial dilution of the transformants mixtures (1, 10^−1^ and 10^−2^). **(B)** SAP binds KIX8 and KIX9 *in vitro*. His-KIX8 and His-KIX9 were incubated with GST-SAP and pulled down by glutathione sepharose. The interactions were detected by immunoblotting with an anti-His antibody. GST-GUS was used as a negative control. **(C)** The split luciferase complementation assays show that SAP associates with KIX8/9 in *N*. *benthamiana*. SAP-nLUC and cLUC-KIX8/9 were co-expressed in *N*. *benthamiana* leaves. Luciferase activity was detected 24 hours after infiltration. The pseudocolor bar represents the range of luminescence intensity in each image. **(D)** SAP interacts with KIX8 and KIX9 in *Arabidopsis*. GFP-Trap-A beads were incubated with total protein extracts of *35S*:*GFP;35S*:*Myc-KIX8*, *35S*:*GFP-SAP;35S*:*Myc-KIX8*, *35S*:*GFP;35S*:*Myc-KIX9* and *35S*:*GFP-SAP;35S*:*Myc-KIX9* transgenic plants, respectively. The interactions were analyzed by immunoblot with anti-Myc or anti-GFP antibodies.

To identify novel components involved in SAP-mediated organ size control, we carried out a yeast two-hybrid screen for SAP-interacting proteins. KIX8 and KIX9 were found to interact with SAP in this screen. KIX8 and KIX9 have been shown to form a repressor complex with TOPLESS and control meristemoid cell proliferation[12], suggesting that KIX8 and KIX9 are good candidates for SAP-interacting proteins. We confirmed that SAP can interact with full-length KIX8 and KIX9 in yeast cells (Fig 1A). To analyze which domain of KIX proteins is responsible for the interaction with SAP, we used different truncations of KIX proteins in the yeast two-hybrid assays. However, none of these truncations showed an interaction with SAP in yeast cells, indicating that full length of KIX proteins is required for the interaction (S1 Fig). To examine their interactions *in vitro*, we performed pull-down assays using His-tagged KIX8 and KIX9 and GST-tagged SAP proteins expressed in *E*. *coli*. As shown in Fig 1B, His-KIX8 and His-KIX9 bound to GST-SAP but not the GST-GUS control, indicating that SAP directly interacts with KIX8 and KIX9 *in vitro*.

We then tested whether SAP could interact with KIX8 and KIX9 *in planta* using split luciferase complementation assays. *Nicotiana benthamiana* leaves cotransformed with *SAP-nLUC* and *cLUC-KIX8* or *cLUC-KIX9* constructs showed luciferase activity, whereas the negative control did not have luciferase activity, indicating that SAP associates with KIX8 and KIX9 *in vivo* (Fig 1C). To confirm the interaction of SAP with KIX8/9, we generated *Arabidopsis* transgenic lines expressing *35S*:*Myc-KIX8* and *35S*:*Myc-KIX9*, and crossed them with *35S*:*GFP-SAP* or *35S*:*GFP* to obtain *35S*:*Myc-KIX8/9* in *35S*:*GFP-SAP* background and the *35S*:*GFP* background, respectively. Co-immunoprecipitation analysis showed that GFP-SAP associated with Myc-KIX8/9 in *Arabidopsis* (Fig 1D). Taken together, these data demonstrate that SAP can form a protein complex with KIX8/9 in *Arabidopsis*.

### SAP regulates the stability of KIX8 and KIX9 proteins

**Fig 2:**
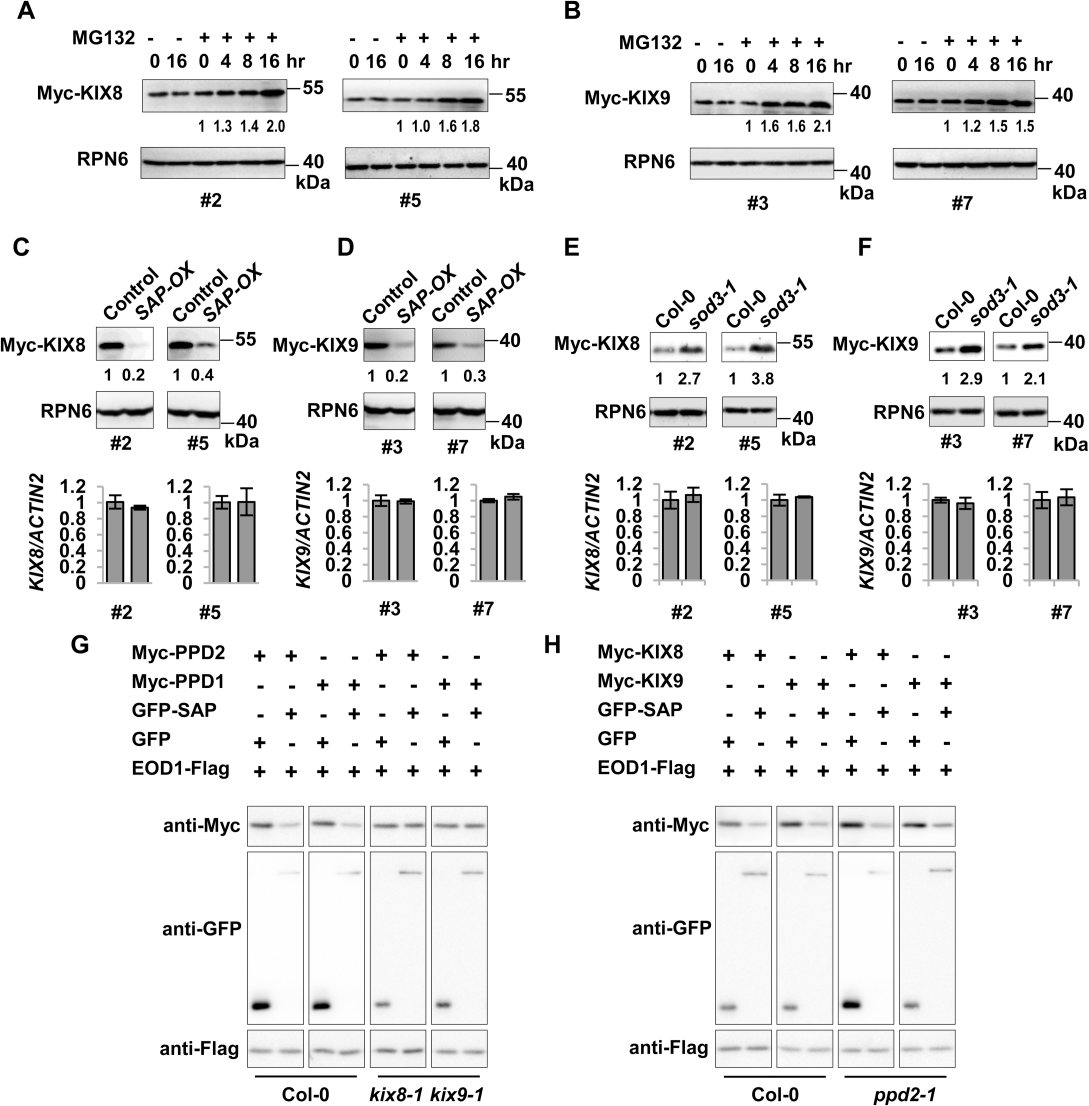
SAP regulates the stability of KIX8 and KIX9. **(A-B)** The proteasome inhibitor MG132 stabilizes KIX8 (**A**) and KIX9 (**B**). Ten-day-old *35S*:*Myc-KIX8* or *35S*:*Myc-KIX9* seedlings were incubated with 50 μM MG132 (+) or DMSO control (-) for 0, 4, 8 and 16 hours. Myc-KIX8 and Myc-KIX9 were detected by immunoblot with anti-Myc antibody. Immunoblot analysis using anti-RPN6 antibody was used as loading controls. Two independent lines of *35S*:*Myc-KIX8* (#2 and #5) and *35S*:*Myc-KIX9* (#3 and #7) were analyzed. **(C-D)** The amounts of KIX8 and KIX9 proteins were decreased in plants overexpressing *SAP*. *35S*:*Myc-KIX8* (#2 and #5) and *35S*:*Myc-KIX9* (#3 and #7) transgenic lines were crossed with *35S*:*GFP* (Control) and *35S*:*GFP-SAP* (*SAP-OX*), respectively. The amounts of Myc-KIX8 or Myc-KIX9 in *35S*:*GFP;35S*:*Myc-KIX8* (Control), *35S*:*GFP-SAP;35S*:*Myc-KIX8* (*SAP-OX*), *35S*:*GFP;35S*:*Myc-KIX9* (Control), and *35S*:*GFP-SAP;35S*:*Myc-KIX9* (*SAP-OX*) was analyzed with anti-Myc antibody. Immunoblot analysis using anti-RPN6 antibody was used as loading controls. Relative transcription levels of *KIX8* and *KIX9* in each line were shown at the bottom. **(E-F)** The amounts of KIX8 and KIX9 proteins were increased in the *sod3-1* mutant. Two independent lines of *35S*:*Myc-KIX8* (#2 and #5) and *35S*:*Myc-KIX9* (#3 and #7) were crossed with *sod3-1*, respectively. The amounts of Myc-KIX8 or Myc-KIX9 in *35S*: *Myc-KIX8*, *35S*:*Myc-KIX8;sod3-1*, *35S*:*Myc-KIX9*, *35S*:*Myc-KIX9;sod3-1* seedlings were analyzed by immunoblot using anti-Myc antibody. Immunoblot analysis using anti-RPN6 antibody was used as loading controls. Relative transcription levels of *KIX8* and *KIX9* in each line were shown at the bottom. Myc-KIX8 and Myc-KIX9 protein levels in **(A)** to **(F)** were quantified by the ImageJ program, and relative levels of Myc-KIX8 and Myc-KIX9 were shown blow the blots. **(G)** SAP-mediated degradation of PPD is dependent of KIX8/9. Myc-PPD1/2 and GFP-SAP or GFP control were co-expressed in Col-0 or *kix8-1 kix9-1* protoplasts, and the amounts of PPD proteins were detected by immunoblot using anti-Myc antibody. EOD1-Flag was used as a control for protoplast transformation. Triplicate transformation evens were performed and representative results were shown. **(H)** The *ppd2-1* mutation did not affect SAP-mediated degradation of KIX8 and KIX9. Myc-KIX8/9 and GFP-SAP or GFP control were co-expressed in Col-0 or *ppd2* protoplasts, and the amounts of KIX proteins were detected by immunoblot using anti-Myc antibody. EOD1-Flag was used as a control for protoplast transformation. Triplicate transformation evens were performed and representative results were shown.

As SAP functions in an SKP1/Cullin/F-box (SCF) E3 ubiquitin ligase complex to mediate proteasome-dependent degradation of substrate proteins[38], we further investigated whether SAP could influence the stability of KIX8 and KIX9. We first tested whether the levels of KIX8 and KIX9 proteins could be affected by the ubiquitin-proteasome system. *35S*:*Myc-KIX8* or *35S*:*Myc-KIX9* transgenic plants were treated with the proteasome inhibitor MG132, and Myc-KIX8 and Myc-KIX9 proteins were then detected by immunoblot analysis. The amounts of Myc-KIX8 and Myc-KIX9 proteins were accumulated during MG132 treatment (Fig 2A and 2B), suggesting that the stability of KIX8 and KIX9 proteins is influenced by the ubiquitin-proteasome pathway.

Next, we checked whether overexpression of *SAP* could affect the protein levels of KIX8 and KIX9. Two independent lines of *35S*:*Myc-KIX8* or *35S*:*Myc-KIX9* were crossed with *35S*:*GFP-SAP* (*SAP-OX*) or *35S*:*GFP* (control) to generate *35S*:*Myc-KIX8/9* in *SAP-OX* background and *35S*:*GFP* (control) background, respectively. Protein extracts of ten-day-old seedlings were subjected to immunoblot analysis. As shown in Fig 2C and 2D, the protein levels of Myc-KIX8 and Myc-KIX9 were obviously decreased in *35S*:*GFP-SAP* lines (*SAP-OX*) compared with those in *35S*:*GFP* lines (Control), while the transcription levels of *KIX8* and *KIX9* were not affected by overexpression of *SAP*, as shown by quantitative real-time PCR analysis (Fig 2C and 2D). These results indicate that overexpression of *SAP* causes the degradation of KIX8 and KIX9 proteins in *Arabidopsis*.

To analyze whether mutations in *SAP* could cause the accumulation of KIX8 and KIX9 in *Arabidopsis*, we crossed two independent lines of *35S*:*Myc-KIX8* and *35S*:*Myc-KIX9* with the *sod3-1* mutant that has a loss-of-function mutation in *SAP* and obtained *35S*:*Myc-KIX8/9* in *sod3-1* background. The *sod3-1* mutant had higher levels of Myc-KIX8 and Myc-KIX9 proteins than the wild type, whereas the transcription levels of *KIX8* and *KIX9* were not affected by the *sod3-1* mutation (Fig 2E and 2F). These data reveal that SAP regulates KIX8/9 protein stability in *Arabidopsis*.

Previously we showed that SAP modulates the protein stability of PPD proteins to regulate organ growth [38]. Therefore, we further asked whether SAP-mediated degradation of PPD is dependent of KIX8/9. We then transiently expressed Myc-PPD and GFP-SAP in the mesophyll protoplasts of either the wild type or *kix8-1 kix9-1*. In the wild type, the protein stability of PPD was decreased by overexpression of SAP, whereas in *kix8-1 kix9-1* the protein stability of PPD was not affected by SAP (Fig 2G). These results suggest that KIX8/9 is required for SAP-mediated degradation of PPD. By contrast, SAP promotes KIX degradation in either wild type or *ppd2* (Fig 2H).

As KIX, PPD and TPL form a protein complex [12], and SAP modulates the protein stability of both KIX and PPD, we further asked whether SAP also affects the protein stability of TPL. We then transiently expressed Myc-TPL and GFP-SAP in protoplasts. However, we did not detect an obvious decrease of the protein level of Myc-TPL when overexpressing GFP-SAP, suggesting that SAP may not promote TPL degradation (S2 Fig).

To investigate whether KIX8 and KIX9 affects the expression of *PPD* genes, we analyzed the transcript levels of *PPD* genes in the *kix* mutants. The expression of *PPD2* was slightly increased in the *kix8-1 kix9-1* mutant (S3 Fig), indicating a feed back regulation of this protein complex in transcription level.

### *SAP* genetically interacts with *KIX8* and *KIX9* to control organ growth

**Fig 3:**
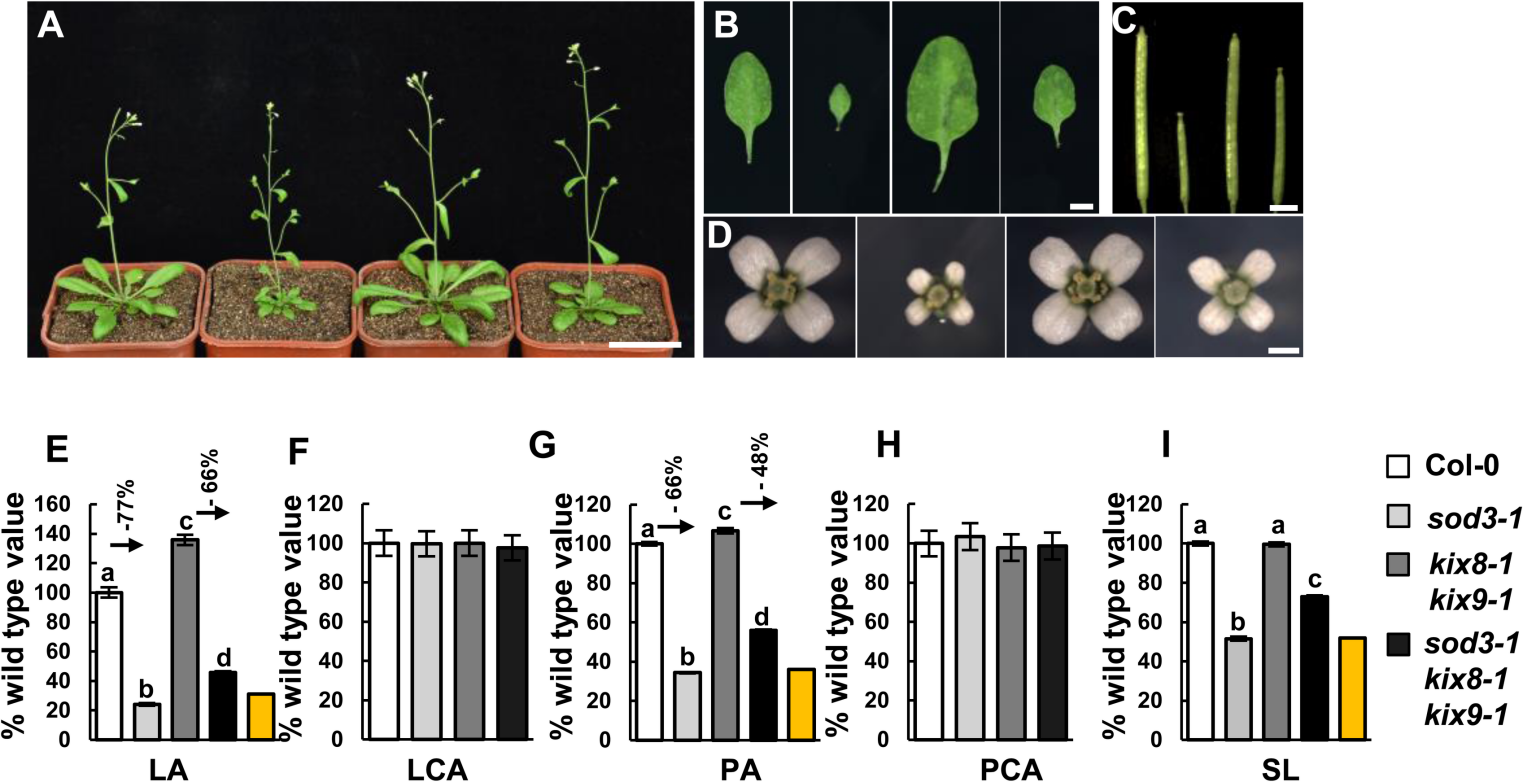
SAP genetically interacts with KIX8 and KIX9 to control organ growth. **(A)** Thirty-day-old plants of Col-0, *sod3-1*, *kix8-1 kix9-1* and *sod3-1 kix8-1 kix9-1* (from left to right). **(B-D)** The fifth leaves (**B**), siliques (**C**) and flowers (**D**) of Col-0, *sod3-1*, *kix8-1 kix9-1* and *sod3-1 kix8-1 kix9-1* (from left to right). (**E-I)** Fifth leaf area (LA), leaf cell area (LCA), petal area (PA), petal cell area (PCA), and silique length (SL) of Col-0, *sod3-1*, *kix8-1 kix9-1* and *sod3-1 kix8-1 kix9-1*. Values in **(E)**-**(I)** are given as mean±s.e. relative to the respective wild-type values, set at 100%. 10 leaves, 300 cells from 10 leaves, 70 petals, 450 cells from 15 petals, and 20 siliques were used to measure LA, LCA, PA, PCA and SL, respectively. The yellow columns indicate the expected LA, PA and SL if *sod3-1* and *kix8-1 kix9-1* have additive effects [expected value = (*kix8-1 kix9-1* value) × (% *sod3-1* value)]. Different lowercase letters above the columns indicate statistically different groups (P <0.01). Scale bars, 5 cm in **(A)**, 5mm in **(B)**, 3mm in **(C)** and 1mm in **(D)**.

As SAP associates with KIX8 and KIX9 and regulates their stability, we investigated whether KIX8 and KIX9 could function with SAP in a common pathway to control organ size. The *kix8-1 kix9-1* double mutant showed large and dome-shaped leaves[12], whereas the *sod3-1* mutant had decreased organ size [38]. We crossed *sod3-1* with *kix8-1 kix9-1* to generate *sod3-1 kix8-1 kix9-1* triple mutant. As shown in Fig 3, the small leaf and flower phenotypes of *sod3-1* were partially suppressed by *kix8-1 kix9-1*. The area of cells in *sod3-1*, *kix8-1 kix9-1* and *sod3-1 kix8-1 kix9-1* leaves and petals was comparable to that in wild-type leaves and petals, suggesting that SAP and KIX control organ growth by influencing cell proliferation (Fig 3F and 3H). Furthermore, the silique length of triple mutant *sod3-1 kix8-1 kix9-1* was significantly increased in comparison with that of *sod3-1*, although *kix8-1 kix9-1* siliques showed similar length to wild-type siliques (Fig 3C and 3I). These genetic data indicate that *kix8-1 kix9-1* is partially epistatic to *sod3-1* with respect to organ size, suggesting that *SAP* functions with *KIX8/9* in a common genetic pathway to control organ growth.

We have previously reported that *35S*:*GFP-SAP* plants have large flowers, dome-shaped leaves and short siliques [38]. By contrast, *35S*:*Myc-KIX8* and *35S*:*Myc-KIX9* plants showed decreased organ size (S4 Fig). The phenotypes of *35S*:*GFP-SAP* were partially rescued by *35S*:*Myc-KIX* (S5 Fig), further suggesting that SAP and KIX8/9 may function in a common genetic pathway to control organ size.

### SAP acts upstream of the KIX-PPD complex to control organ growth

**Fig 4:**
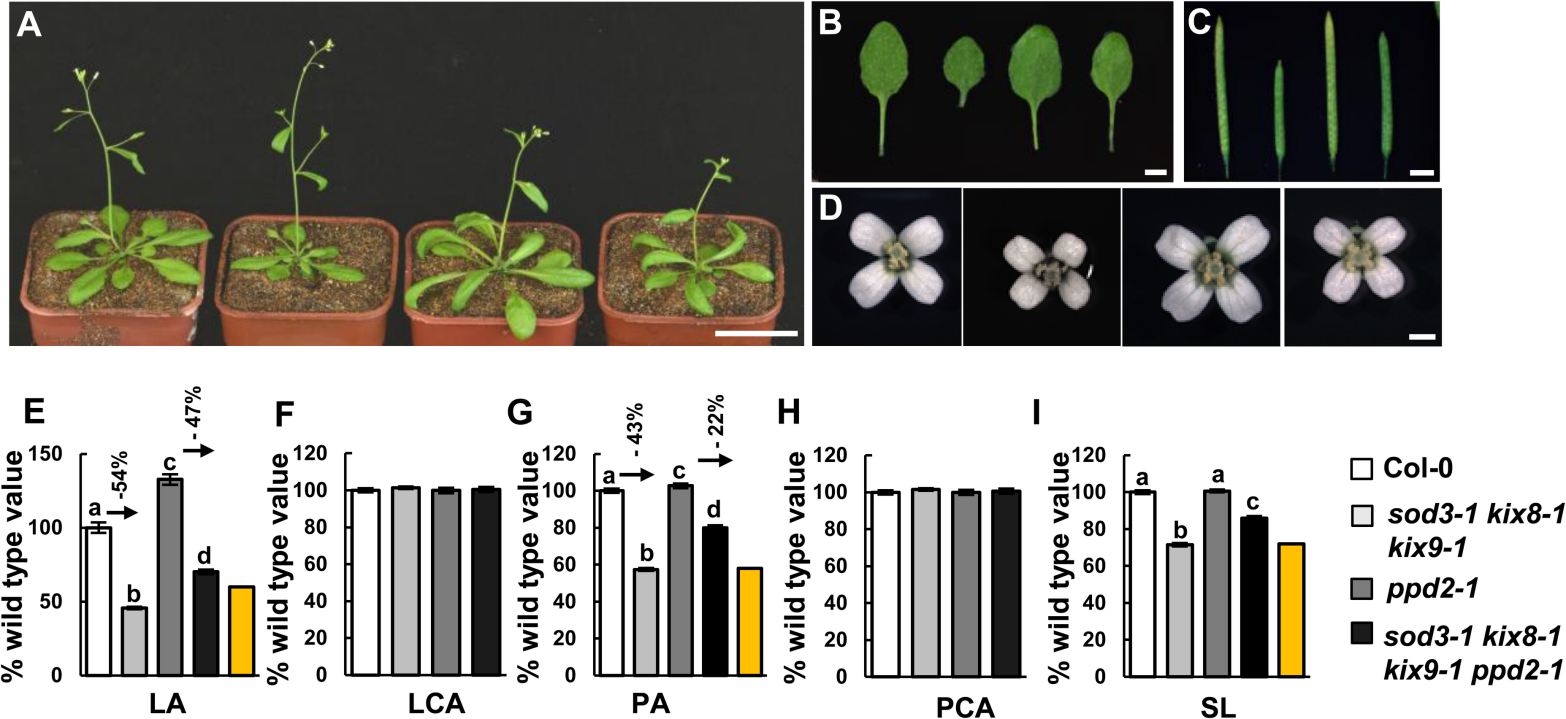
SAP, KIX and PPD act in a common genetic pathway to control organ growth. **(A-D)** Thirty-day-old plants **(A)**, the fifth leaves **(B)**, siliques **(C)** and flowers **(D)** of Col-0, *sod3-1 kix8-1 kix9-1*, *ppd2-1*, and *sod3-1 kix8-1 kix9-1 ppd2-1* (from left to right). **(E-I)** Fifth leaf area (LA), leaf cell area (LCA), petal area (PA), petal cell area (PCA), and silique length (SL) of Col-0, *sod3-1 kix8-1 kix9-1*, *ppd2-1*, and *sod3-1 kix8-1 kix9-1 ppd2-1*. Values **(E-I)** in are given as mean±s.e. relative to the respective wild-type values, set at 100%. 10 leaves, 300 cells from 10 leaves, 70 petals, 450 cells from 15 petals, and 20 siliques were used to measure LA, LCA, PA, PCA, and SL, respectively. The yellow columns indicate the expected LA, PA and SL if *sod3-1 kix8-1 kix9-1* and *ppd2-1* have additive effects [expected value = (*sod3-1 kix8-1 kix9-1* value) × (% *ppd2-1* value)]. Different lowercase letters above the columns indicate statistically different groups (P <0.01). Scale bars, 5cm in **(A)**, 5mm in **(B)**, 3mm in **(C)** and 1mm in **(D)**.

We have previously shown that SAP associates with PPD proteins and regulates their stability to control organ growth[38]. SAP acts genetically with PPD to regulate organ growth[38]. It has been reported that PPD proteins physically interact with KIX8 and KIX9 [12], although their genetic interactions remain unknown so far. We therefore asked whether SAP, KIX8, KIX9 and PPD could act in a common genetic pathway to control organ size. The *Δppd* mutant (L*er* ecotype) showed large and dome-shape leaves due to the deletion of both *PPD1* and *PPD2* genes [8]. In Columbia ecotype (Col-0), *ppd2-1* and *ami-ppd* showed large and dome-shape leaves, like those observed in the *Δppd* mutant [8, 12, 38]. As *sod3-1* and *kix8-1 kix9-1* mutants are in Col-0, we conducted a cross between *sod3-1 kix8-1 kix9-1* and *ppd2-1* to generate the quadruple mutant *sod3-1 kix8-1 kix9-1 ppd2-1*. As shown in Fig 4, the leaf, petal and silique phenotypes of *sod3-1 kix8-1 kix9-1* triple mutant were partially suppressed by *ppd2-1* (Fig 4). It was shown that KIX8 and KIX9 interact with PPD[12], and SAP modulates the stability of both PPD and KIX8/9. Thus, it is possible that SAP may act upstream of the KIX-PPD complex to control organ growth.

### The SAP-KIX-PPD module regulates meristemoid cell proliferation

**Fig 5:**
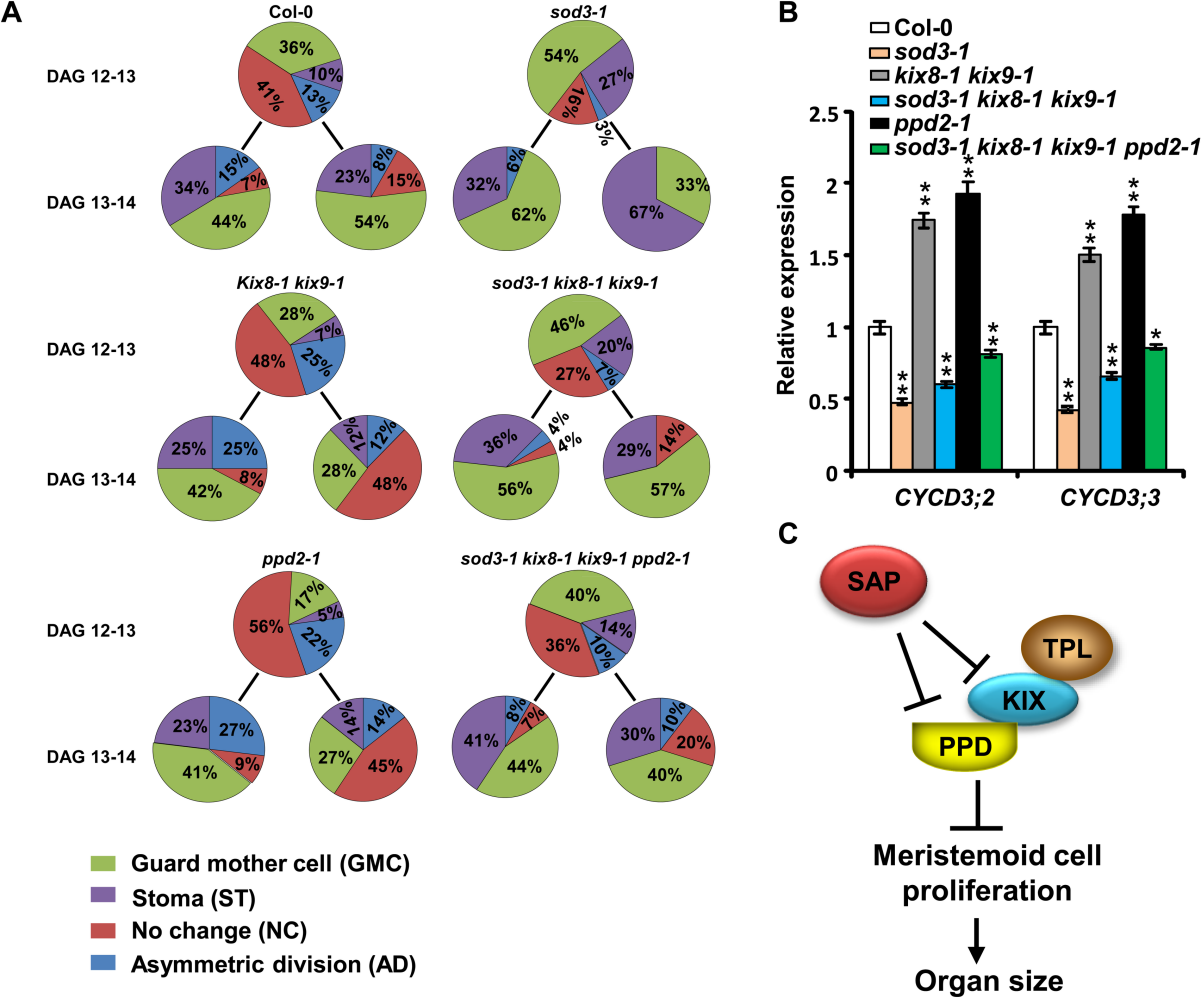
The SAP-KIX-PPD module controls meristemoid cell proliferation. **(A)** Proportion of cell types (meristemoid after asymmetric division, guard mother cell, stoma, or meristemoid) originating from meristemoids were followed from 12 to 14 DAG by making daily dental resin imprints of the abaxial epidermis of leaves. Representative images of dental resin imprints of the abaxial epidermis of leaves at 12 to 14 DAG used to estimate the proportion of cell types originating from the meristemoid division were shown in S1 Fig. **(B)** Relative expression levels of *CYCD3;2* and *CYCD3;3* in the first pair of leaves of 12-d-old Col-0, *sod3-1*, *kix8-1 kix9-1*, *sod3-1 kix8-1 kix9-1*, *ppd2-1*, and *sod3-1 kix8-1 kix9-1 ppd2-1* seedlings. *P<0.05, **P<0.01 compared with the wild type (Student’s t-test). **(C)** A genetic and molecular framework for SAP, KIX and PPD-mediated regulation of meristemoid cell proliferation and organ size. The PPD-KIX-TPL complex controls organ growth by restricting meristemoid proliferation. SAP promotes meristemoid proliferation by targeting KIX and PPD proteins for degradation.

To analyze how the SAP-KIX-PPD module regulates meristemoid cell proliferation, we performed dental resin imprints of the leaf epidermis to follow the fate of meristemoid cells from 12 to 14 days after germination (DAG). Meristemoids are small triangular cells originating from asymmetric division of meristemoid mother cells. A meristemoid undergoes limited rounds of asymmetric divisions before it becomes a guard mother cell or a stoma[11, 13, 39]. We investigated how many meristemoid cells become guard mother cells and stomas or undergo asymmetric division and still maintain the meristemoid functions over time. In the *sod3-1* mutant, the proportion of asymmetric dividing meristemoids was decreased compared with that in the wild type between 12 and 13 DAG (3% versus 13%) and between 13 and 14 DAG (6%versus 15%) (Figs 5A and S6). By contrast, more meristemoids in *sod3-1* became guard mother cells or stomata than those in the wild type (Fig 5A). These results indicate that meristemoid division in *sod3-1* arrests earlier than that in the wild type. In contrast, the *kix8-1 kix9-1* mutant and the *ppd2-1* mutant showed more amplifying division of meristemoid cells than the wild type (Fig 5A), which is consistent with previous studies[8, 12]. The *sod3-1 kix8-1 kix9-1* triple mutant showed an increased proportion of asymmetric dividing cells and a decreased proportion of meristemoids becoming guard mother cells or stomata compared with the *sod3-1* single mutant, indicating that *kix8 kix9* partially suppresses the arrested proliferation of meristemoids in *sod3-1* (Fig 5A). Furthermore, more meristemoids in the *sod3-1 kix8-1 kix9-1 ppd2-1* quadruple mutant underwent asymmetric division than those in the *sod3-1 kix8-1 kix9-1* triple mutant. These results suggest that *SAP* functions genetically with *KIX8*, *KIX9* and *PPD2* to control meristemoid cell proliferation.

### The SAP-KIX-PPD module regulates the expression of PPD target genes

The PPD proteins have been reported to associate with the promoter of their target genes such as *CYCD3;2* and *CYCD3;3* and repress their expression[12]. KIX8 and KIX9 form the complex with PPD and are required for the repression of PPD target genes[12]. As SAP controls meristemoid proliferation and organ growth by targeting PPD and KIX for degradation, we investigated whether SAP could influence the expression of PPD target genes. We analyzed the mRNA levels of *CYCD3;2* and *CYCD3;3* in the first pair of leaves at 12 DAG. As shown in Fig 5B, expression levels of *CYCD3;2* and *CYCD3;3* were higher in the *kix8-1 kix9-1* and the *ppd2-1* mutants than those in the wild type, which is consistent with the previous study[12] (Fig 5B). By contrast, expression of *CYCD3;2* and *CYCD3;3* was down-regulated in the *sod3-1* mutant, suggesting that SAP positively influences the expression of these two genes (Fig 5B). In accordance with the organ size phenotypes, *kix8-1 kix9-1* partially suppressed the reduced expression levels of *CYCD3;2* and *CYCD3;3* in *sod3-1*. Similarly, expression levels of *CYCD3;2* and *CYCD3;3* in *sod3-1 kix8-1 kix9-1 ppd2-1* were higher than those in *sod3-1 kix8-1 kix9-1*. Thus, these results suggest that SAP acts upstream of the KIX-PPD complex to regulate the expression of PPD target genes. In addition, expression levels of several other PPD-regulated genes related to cell proliferation and organ growth were also suppressed in the *sod3-1* mutant background, and the suppression was partially released by *kix8-1 kix9-1* and *ppd2-1* (S7 Fig). These data further support that the SAP-KIX-PPD module regulates the expression of common downstream genes to regulate organ growth.

## Discussion

How plants determine their organ size is an interesting part of developmental biology. Meristemoids, which possess stem cell-like activity, have been recognized to regulate organ growth in *Arabidopsis*. However, only a few factors have been described to regulate organ growth through meristemoid cell proliferation. We have previously demonstrated that SAP promotes organ growth by increasing meristemoid cell proliferation. SAP mediates the degradation of PPD1 and PPD2 [38], two negative regulators of meristemoid cell proliferation[8]. In this study, we identify KIX8 and KIX9 as two novel targets of SAP. SAP directly interacts with KIX8 and KIX9, and modulates their protein stability. Genetic analyses suggest that *SAP* functions with *KIX8* and *KIX9* in a common pathway to regulate organ growth through meristemoid cell proliferation. Our results reveal a novel molecular mechanism that SAP targets the KIX-PPD complex for degradation to control meristemoid cell proliferation and organ growth.

In *Arabidopsis* leaves, meristemoid cell division gives rise to almost half of the total number of pavement cells, thereby contributing significantly to the final leaf size[9, 10]. The PPD proteins were the first two identified factors that control organ size by restricting meristemoid cell proliferation in *Arabidopsis*[8]. The *ppd* mutants showed large and dome-shaped leaves due to increased meristemoid cell proliferation. The KIX8 and KIX9 have been recently shown to recruit the transcription repressor TPL and form a repressor complex with PPD[12]. The *kix8-1 kix9-1* mutant exhibited similar organ growth phenotypes to *ppd* mutants, although their genetic interactions remain unknown. We have recently revealed that SAP/SOD3, an F-box protein, modulates the stability of PPD to control organ growth by influencing meristemoid cell proliferation[38]. Considering that the *ppd* mutant only partially suppressed the organ growth phenotypes of *sod3-1*[38], it is possible that SAP may target other substrates for degradation as well. Supporting this idea, we demonstrate that SAP interacts with KIX8 and KIX9 and targets them for degradation. Overexpression of *SAP* resulted in the destabilization of KIX8 and KIX9 proteins, while KIX8 and KIX9 proteins were accumulated in the *sod3-1* mutant compared with those in the wild type. Interestingly, SAP-mediated degradation of PPD proteins is dependent of KIX8 and KIX9, indicating that SAP may target PPD-KIX complex for degradation. Genetic analyses showed that *kix8-1 kix9-1* partially suppressed the organ growth and meristemoid proliferation phenotypes of *sod3-1*, suggesting that *SAP* may act in a common pathway with *KIX8* and *KIX9* to control organ growth by regulating meristemoid cell proliferation. In addition, overexpression of *KIX8* or *KIX9* decreases organ size, and *35S*:*Myc-KIX8* or *35S*:*Myc-KIX9* can partially rescue the organ size phenotypes of *35S*:*GFP-SAP*, reinforcing the genetic interaction of *SAP* and *KIX*. These results support that KIX8 and KIX9 are two novel substrates of SAP, and SAP targets the KIX-PPD complex for degradation in *Arabidopsis*. Consistent with this conclusion, the simultaneous disruptions of both *KIX8/9* and *PPD2* suppressed the organ growth and meristemoid proliferation phenotypes of *sod3-1* better than the disruption of either *KIX8/9* or *PPD2*.

It has been shown that the PPD-KIX complex represses the expression of D-type cyclins and other target genes. Loss-of-function of *PPD* or *KIX* results in up-regulation of PPD target genes. Interestingly, we found that PPD target genes, such as *CYCD3;2* and *CYCD3;3*, were down-regulated in the *sod3-1* mutant (Fig 5B), suggesting that SAP positively influences the expression of PPD target genes. The expression levels of *CYCD3;2* and *CYCD3;3* in *sod3-1* were partially rescued by *kix8-1 kix9-1*, and further restored by *kix8-1 kix9-1 ppd2-1* (Fig 5B), suggesting that SAP functions with the KIX-PPD complex in a common pathway to regulate the expression of PPD target genes. Thus, it is possible that SAP may release the transcriptional repression of PPD target genes by targeting the KIX-PPD repressor complex for degradation (Fig 5C). Notably, in *sod3-1 ppd2-1 kix8 kix9*, the expression levels of CYCD3;2 and CYCD3;3 are still decreased compared to the wild type, which is consistent with the observation that the organ size of *sod3-1 ppd2-1 kix8 kix9* is decreased compared to the wild type. This indicates that SAP may target other substrates which also regulate the expression of CYCD3;2 and CYCD3;3. We have shown that SAP medicates the degradation of PPD1, which functions redundantly with PPD2 in organ size control [38]. Besides, SAP may target other unidentified substrates, including KIX8/9 homologues, for degradation. In *sod3-1 ppd2-1 kix8 kix9*, PPD1 and other SAP targets may accumulate and repress the expression of CYCD3;2 and CYCD3;3.

Organ size is an important agronomic trait that influences biomass and yield. Leaves or seeds are usually harvested as the main products in crops. Thus, the increased production of plant organs would be valuable for crop producers. The *ppd* and *kix* mutants produced large organs, and overexpression of *SAP* increased organ size in *Arabidopsis*. Interestingly, the *mtbs-1* mutant, which contains a mutation in the *PPD* homolog, has been recently reported to produce large leaves, seed pods and seeds in *Medicago truncatula*[40]. Down-regulation of the *PPD* homolog can also increase seed size and quality in soybean[40]. In addition, a recent study showed that allelic variation in the intron of *SAP* homolog contributes to flower size in *Capsella*[41]. These studies suggest that the SAP-KIX-PPD module have conserved functions in different plant species. As homologs of *SAP*, *KIX* and *PPD* are found in eudicot genera[8, 12, 38], *SAP*, *KIX* and *PPD* homologs in dicots (e.g soybean and oilseed rape) could be manipulated to increase seed and organ size in crops. During breeding programs, breeders have already selected important yield related traits, such as seed and organ size and seed shape. Thus, it will be interesting to investigate whether natural allelic variations of *SAP*, *KIX* and *PPD* homologs have been selected by crop breeders in the future.

## Methods

### Plant materials and growth conditions

The *suppressor of da1-1* (*sod3-1*), *ppd2-1* (SALK_142698), *35S*:*GFP-SAP*, *35S*:*GFP*, and *kix8-1 kix9-1* plants were described previously[12, 38]. All transgenic plants and mutants were in the *Arabidopsis thaliana* Col-0 ecotype. Plants were grown in greenhouse under the long-day conditions (16 hrs light/8 hrs dark) at 22°C.

### Constructs and plant transformation

The primers used for all the constructs were listed in S1 Table. The coding sequences (CDS) of *KIX8* and *KIX9* were cloned into *pCAMBIA1300-221-Myc* to construct *35S*:*Myc-KIX8/9*. The plasmids were transformed into *Arabidopsis* plants using *Agrobacterium tumefaciens* GV3101. MS medium supplemented with hygromycin (30 μg ml^−1^) was used to screen transformants. T2 seeds with a typical 3:1 segregation ratio for hygromycin-resistant versus hygromycin-sensitive were used for protein stability analysis.

### Morphological and cellular analysis

To measure leaf area, petal area and silique length, we photographed leaves, petals (stage 14) and siliques and used ImageJ software to analyze the images. To measure cell size, leaves and petals were treated with the clearing solution [38] and then photographed under a differential interference contrast (DIC) microscope (Leica DM2500). The middle region of adaxial side of petals and the palisade parenchyma cells in the middle region of the leaves were used for cell size measurement.

Dental resin imprints were performed as described before[13]. The dental resin imprints were taken daily from the abaxial surface of the first leaves from DAG (day after germination) 12 to DAG 14. The surface of the epidermis was copied with Vinyl Polysiloxane impression material. The Vinyl Polysiloxane impression surface was further copied by covering with clear nail polish, and the nail polish copies were observed by scanning electron microscopy.

### Quantitative Real Time -PCR (RT-PCR) analysis

The primers used for quantitative RT-PCR analysis were listed in S1 Table. Total RNA extraction and quantitative RT-PCR analysis were performed as described before [38]. *ACTIN2* was used as a control for normalization. Relative amounts of mRNA were calculated using the Cycle threshold (Ct) method as described previously[38].

### Yeast assays

For yeast two-hybrid analysis, the bait construct *pGBKT7-SAP* described before[38] was used to screen for SAP interacting proteins using the Matchmaker Gold Yeast Two-Hybrid system (Clontech). The CDS of *KIX8* and *KIX9* were cloned into *pGADT7* to confirm the interaction of KIX8/9 with SAP. The primers used to construct *pGADT7-KIX8/9* were listed in S1 Table. Transformation of yeast cells was performed according to the user manual (Clontech). Transformation of the bait vector *pGBKT7* with *KIX8-AD* and KIX9*-AD* and the prey vector *pGADT7* with *SAP-BD* was used as the negative control.

### Arabidopsis protoplast isolation and transformation

Protoplasts were isolated from *Arabidopsis* leaves and the transformation was performed as described before [42]. EOD1-FLAG was used as a control for protoplast transformation to indicate that transformation efficiency was comparable between different transformations. After transformation, protoplasts were cultured for 16 hours at 22°C in the dark and then total proteins were isolated for Western blot analysis.

### *In vitro* protein-protein interaction

The primers used to construct *His-KIX8/9* were listed in S1 Table. The coding sequences of *KIX8* and *KIX9* were cloned into *pET-28a(*+*)*. *GST-SAP* and *GST-GUS* were described before[38]. Pull-down assay was performed as described previously[27], and the precipitates were analyzed by immunoblot with anti-GST (Abmart) and anti-His (Abmart) antibodies.

### Split luciferase complementation assay

The primers used to construct *cLUC-KIX8/9* and *SAP-nLUC* were listed in S1 Table. The CDS of *KIX8* and *KIX9* were cloned into the vector *pCAMBIA-split_cLUC*, and the CDS of *SAP* was cloned into the vector *pCAMBIA-split_nLUC*. The plasmids were transformed into *A*. *tumefaciens* GV3101 and transiently expressed in *N*. *benthamiana* leaves as described previously[27]. The luciferin (0.5 mM) was sprayed on leaves and incubated 3 min before luminescence detection by NightOWL II LB983 imaging apparatus.

### *In vivo* co-immunoprecipitation

To prevent protein degradation, seedlings were pre-treated with MG132 before co-immunoprecipitation experiments. Co-immunoprecipitation was performed as described before [27]. The immunoprecipitates were detected by immunoblot analysis with anti-Myc (Abmart) and anti-GFP (Abmart) antibodies, respectively.

### MG132 treatment and protein stability analysis

Ten-day-old seedlings were incubated in liquid MS medium with 50 μM MG132 or DMSO control for 16 h. Total protein were extracted and analyzed by immunoblot using anti-RPN6 (Enzo) and anti-Myc (Abmart) antibodies, respectively. Myc-KIX8 and Myc-KIX9 protein levels were quantified by ImageJ software.

### Accession numbers

Sequence data from this article can be found in the EMBL/GenBank data libraries under accession numbers: AT5G35770 (STERILE APETALA [SAP]), AT4G14713 (PEAPOD1 [PPD1]), AT4G14720 (PEAPOD2 [PPD2]), AT3G24150 (KIX8), and AT4G32295 (KIX9).

## Supporting information

S1 Fig SAP interacts with KIX8 and KIX9 in yeast cells. (A) SAP interacts with full-length KIX8 and KIX9, but does not interact with the truncations of KIX proteins in yeast cells. Transformants were selected on media -2 (SD/-Leu/-Trp), and interactions were tested on media -4 (SD/-Ade/-His/-Leu/-Trp) using a serial dilution of the transformants mixtures (1, 10^-1^and 10^−2^). (B) Schematic diagram of KIX8/9 and the derivatives containing specific protein domains.
(PDF)

S2 Fig The protein level of TPL is not affected by overexpression of SAP. Myc-TPL and GFP-SAP or GFP control were co-expressed in Col-0 protoplasts, and the amount of TPL proteins was detected by immunoblot using anti-Myc antibody. EOD1-Flag was used as a control for protoplast transformation.
(PDF)

S3 Fig Expression of *PPD* in the *kix* mutants. *P<0.05 compared with the wild type (Student’s t-test).
(PDF)

S4 Fig Organ size phenotypes of *35S*: *Myc-KIX* plants. (A-D) The thirty-day-old plants (A), fifth leaves (B), siliques (C) and flowers (D) of Col-0, *35S*: *Myc-KIX8 #2*, *35S*: *Myc-KIX8 #5*, *35S*: *Myc-KIX9 #3*, and *35S*: *Myc-KIX9 #7* (from left to right). (E) Expression of Myc-KIX proteins in the transgenic plants showing by western blot. 1, Col-0, 2, *35S*: *Myc-KIX8 #2*, *3*, *35S*: *Myc-KIX8 #5*, *4*, *35S*: *Myc-KIX9 #3*, 5, *35S*: *Myc-KIX9 #7* (F-J) Fifth leaf area (LA), leaf cell area (LCA), petal area (PA), petal cell area (PCA), and silique length (SL) of Col-0, *35S*: *Myc-KIX8 #2*, *35S*: *Myc-KIX8 #5*, *35S*: *Myc-KIX9 #3*, and *35S*: *Myc-KIX9 #7*. Values are given as mean±s.e. relative to the respective wild-type values, set at 100%. 10 leaves, 70 petals, and 20 siliques were used to measure LA, PA, and SL, respectively. 10 leaves and 15 petals were used to measure LCA and PCA, respectively. **P<0.01 compared with the wild type (Student’s t-test). Scale bars, 5cm in (A), 5mm in (B), 3mm in (C) and 1mm in (D).
(PDF)

S5 Fig Organ size phenotypes of Col-0, *35S*: *GFP-SAP*, *35S*: *Myc-KIX8 #2*, *35S*: *GFP-SAP; 35S*: *Myc-KIX8 #2*, *35S*: *Myc-KIX9 #3*, *35S*: *GFP-SAP; 35S*: *Myc-KIX9 #3* plants. (A-D) The thirty-day-old plants (A), fifth leaves (B), siliques (C) and flowers (D) of Col-0, *35S*: *GFP-SAP*, *35S*: *Myc-KIX8 #2*, *35S*: *GFP-SAP; 35S*: *Myc-KIX8 #2*, *35S*: *Myc-KIX9 #3*, *35S*: *GFP-SAP; 35S*: *Myc-KIX9 #3* (from left to right). (E) Expression of Myc-KIX proteins in different genetic background showing by western blot. 1, Col-0, 2, *35S*: *GFP-SAP*, *3*, *35S*: *Myc-KIX8 #2*, *4*, *35S*: *GFP-SAP; 35S*: *Myc-KIX8 #2*, 5, *35S*: *Myc-KIX9 #3*, 6, *35S*: *GFP-SAP; 35S*: *Myc-KIX9 #3* (F-H) Fifth leaf area (LA), petal area (PA), and silique length (SL) of Col-0, *35S*: *GFP-SAP*, *35S*: *Myc-KIX8 #2*, *35S*: *GFP-SAP; 35S*: *Myc-KIX8 #2*, *35S*: *Myc-KIX9 #3*, *35S*: *GFP-SAP; 35S*: *Myc-KIX9 #3*. Values are given as mean±s.e. relative to the respective wild-type values, set at 100%. 10 leaves, 60 petals and 20 siliques were used to measure LA, PA and SL, respectively. Different lowercase letters above the columns indicate statistically different groups (P <0.01). (I) The expression levels of *CYCD3;2* and *CYCD3;3* in Col-0, *35S*: *GFP-SAP*, *35S*: *Myc-KIX8 #2*, *35S*: *GFP-SAP; 35S*: *Myc-KIX8 #2*, *35S*: *Myc-KIX9 #3*, *35S*: *GFP-SAP; 35S*: *Myc-KIX9 #3* plants. * P<0.05; ** P<0.01 compared with the wild type (Student’s t-test). Scale bars, 5cm in (A), 5mm in (B), 3mm in (C) and 1mm in (D).
(PDF)

S6 Fig Representative images of dental resin imprints of the abaxial epidermis of first pair of leaves at 12 to 14 DAG. Meristemoid cells monitored were marked as yellow. Arrows label the asymmetric division of one meristemoid cell. Bar, 50 μm.
(PDF)

S7 Fig Relative expression levels of cell proliferation and organ growth-related genes in the first pair of leaves of twelve-day-old Col-0, *sod3-1*, *kix8-1 kix9-1*, *sod3-1 kix8-1 kix9-1*, *ppd2-1*, and *sod3-1 kix8-1 kix9-1 ppd2-1* seedlings. *P<0.05, **P<0.01 compared with the wild type (Student’s t-test).
(PDF)

S1 Table List of primers used in this study. (PDF)
