# Supplementary figures and images for: STERILE APETALA modulates the stability of a repressor protein complex to control organ size in Arabidopsis thaliana
Source: PLoS Genet. 2018 Feb 5;14(2):e1007218. doi: 10.1371/journal.pgen.1007218 (PMC5814100; doi:10.1371/journal.pgen.1007218)

A

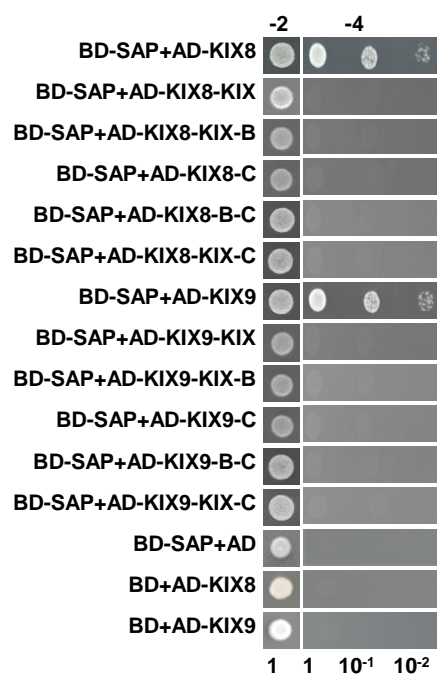

B

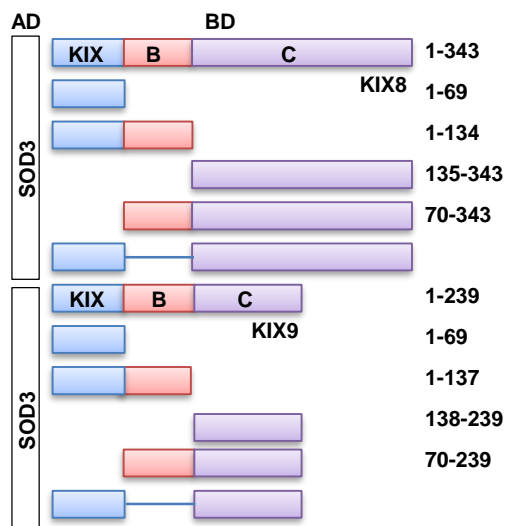

Figure S1

Supplement: S1 Fig — (A) SAP interacts with full-length KIX8 and KIX9, but does not interact with the truncations of KIX proteins in yeast cells. Transformants were selected on media -2 (SD/-Leu/-Trp), and interactions were tested on media -4 (SD/-Ade/-His/-Leu/-Trp) using a serial dilution of the transformants mixtures (1, 10-1and 10−2). (B) Schematic diagram of KIX8/9 and the derivatives containing specific protein domains. (PDF) [file pgen.1007218.s001.pdf]

+ + Myc-TPL  
- + GFP-SAP  
+ - GFP  
+ + EOD1-Flag

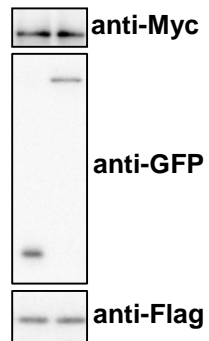

Figure S2

Supplement: S2 Fig — Myc-TPL and GFP-SAP or GFP control were co-expressed in Col-0 protoplasts, and the amount of TPL proteins was detected by immunoblot using anti-Myc antibody. EOD1-Flag was used as a control for protoplast transformation. (PDF) [file pgen.1007218.s002.pdf]

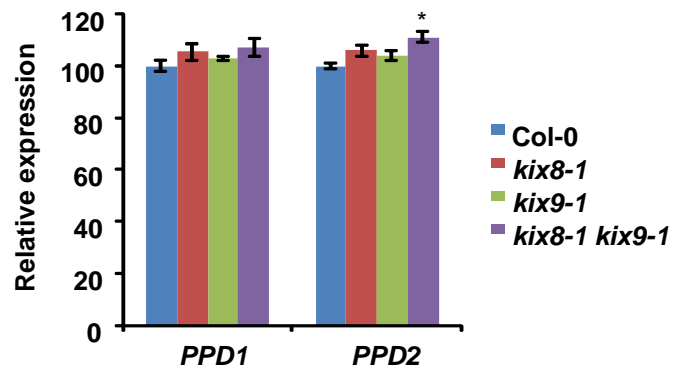

Figure S3

Supplement: S3 Fig — *P<0.05 compared with the wild type (Student’s t-test). (PDF) [file pgen.1007218.s003.pdf]

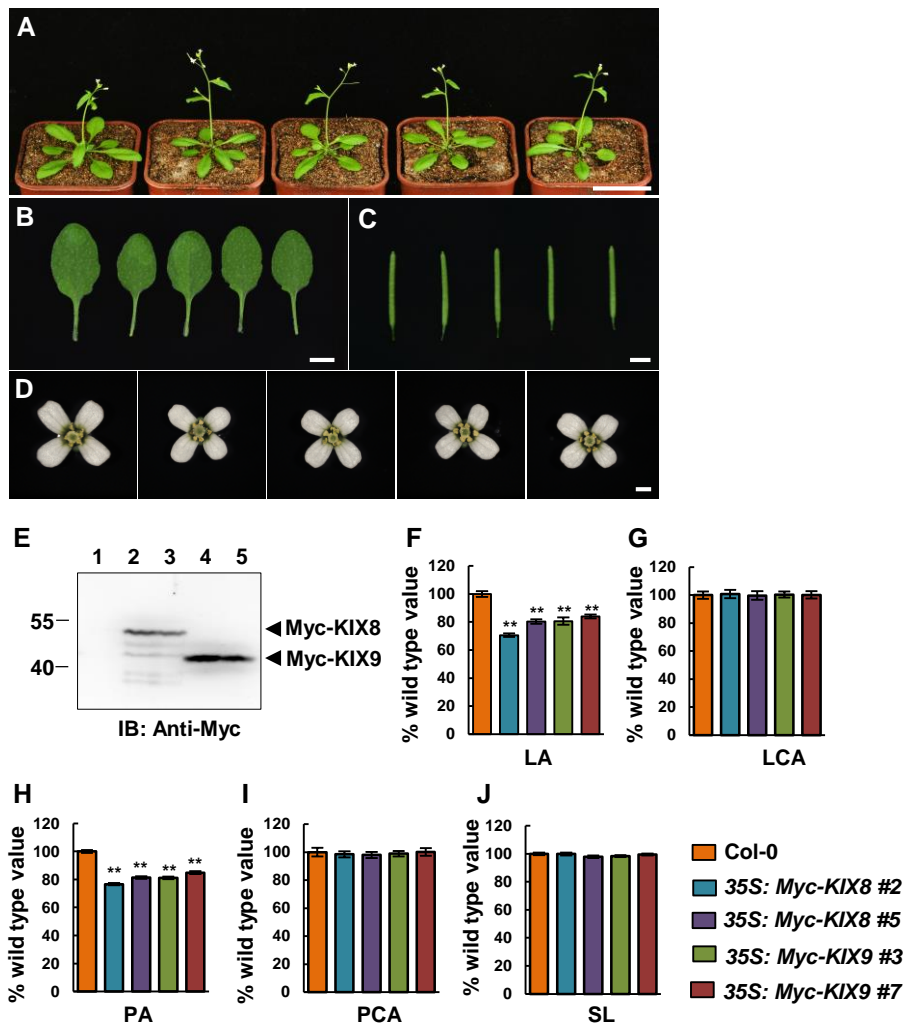

Figure S4

Supplement: S4 Fig — (A-D) The thirty-day-old plants (A), fifth leaves (B), siliques (C) and flowers (D) of Col-0, 35S: Myc-KIX8 #2, 35S: Myc-KIX8 #5, 35S: Myc-KIX9 #3, and 35S: Myc-KIX9 #7 (from left to right). (E) Expression of Myc-KIX proteins in the transgenic plants showing by western blot. 1, Col-0, 2, 35S: Myc-KIX8 #2, 3, 35S: Myc-KIX8 #5, 4, 35S: Myc-KIX9 #3, 5, 35S: Myc-KIX9 #7 (F-J) Fifth leaf area (LA), leaf cell area (LCA), petal area (PA), petal cell area (PCA), and silique length (SL) of Col-0, 35S: Myc-KIX8 #2, 35S: Myc-KIX8 #5, 35S: Myc-KIX9 #3, and 35S: Myc-KIX9 #7. Values are given as mean±s.e. relative to the respective wild-type values, set at 100%. 10 leaves, 70 petals, and 20 siliques were used to measure LA, PA, and SL, respectively. 10 leaves and 15 petals were used to measure LCA and PCA, respectively. **P<0.01 compared with the wild type (Student’s t-test). Scale bars, 5cm in (A), 5mm in (B), 3mm in (C) and 1mm in (D). (PDF) [file pgen.1007218.s004.pdf]

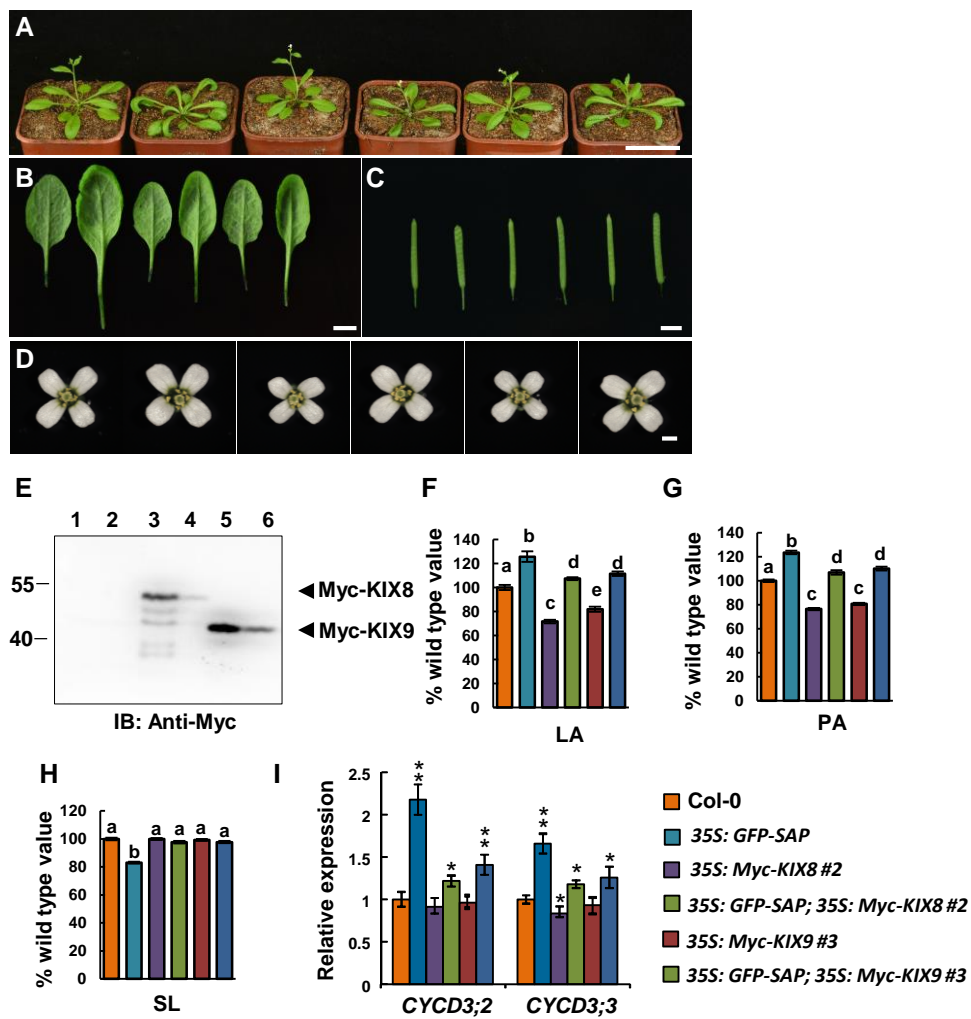

Figure S5

Supplement: S5 Fig — (A-D) The thirty-day-old plants (A), fifth leaves (B), siliques (C) and flowers (D) of Col-0, 35S: GFP-SAP, 35S: Myc-KIX8 #2, 35S: GFP-SAP; 35S: Myc-KIX8 #2, 35S: Myc-KIX9 #3, 35S: GFP-SAP; 35S: Myc-KIX9 #3 (from left to right). (E) Expression of Myc-KIX proteins in different genetic background showing by western blot. 1, Col-0, 2, 35S: GFP-SAP, 3, 35S: Myc-KIX8 #2, 4, 35S: GFP-SAP; 35S: Myc-KIX8 #2, 5, 35S: Myc-KIX9 #3, 6, 35S: GFP-SAP; 35S: Myc-KIX9 #3 (F-H) Fifth leaf area (LA), petal area (PA), and silique length (SL) of Col-0, 35S: GFP-SAP, 35S: Myc-KIX8 #2, 35S: GFP-SAP; 35S: Myc-KIX8 #2, 35S: Myc-KIX9 #3, 35S: GFP-SAP; 35S: Myc-KIX9 #3. Values are given as mean±s.e. relative to the respective wild-type values, set at 100%. 10 leaves, 60 petals and 20 siliques were used to measure LA, PA and SL, respectively. Different lowercase letters above the columns indicate statistically different groups (P <0.01). (I) The expression levels of CYCD3;2 and CYCD3;3 in Col-0, 35S: GFP-SAP, 35S: Myc-KIX8 #2, 35S: GFP-SAP; 35S: Myc-KIX8 #2, 35S: Myc-KIX9 #3, 35S: GFP-SAP; 35S: Myc-KIX9 #3 plants. * P<0.05; ** P<0.01 compared with the wild type (Student’s t-test). Scale bars, 5cm in (A), 5mm in (B), 3mm in (C) and 1mm in (D). (PDF) [file pgen.1007218.s005.pdf]

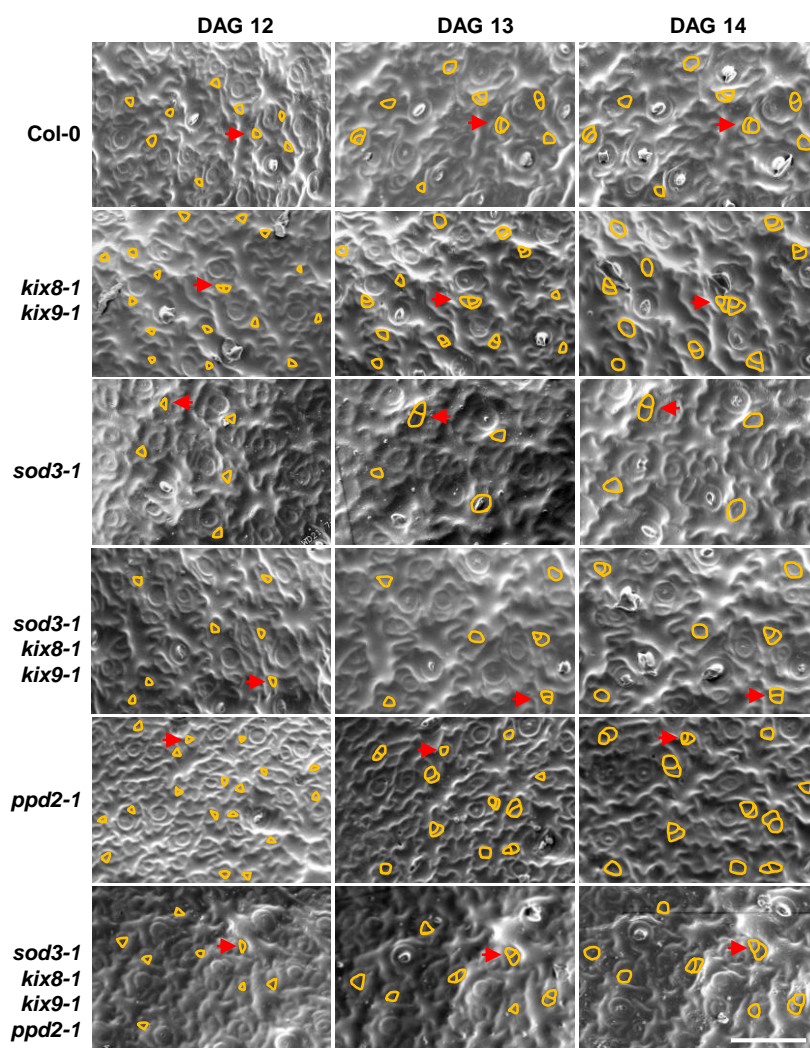

**Figure S6**

Supplement: S6 Fig — Meristemoid cells monitored were marked as yellow. Arrows label the asymmetric division of one meristemoid cell. Bar, 50 μm. (PDF) [file pgen.1007218.s006.pdf]

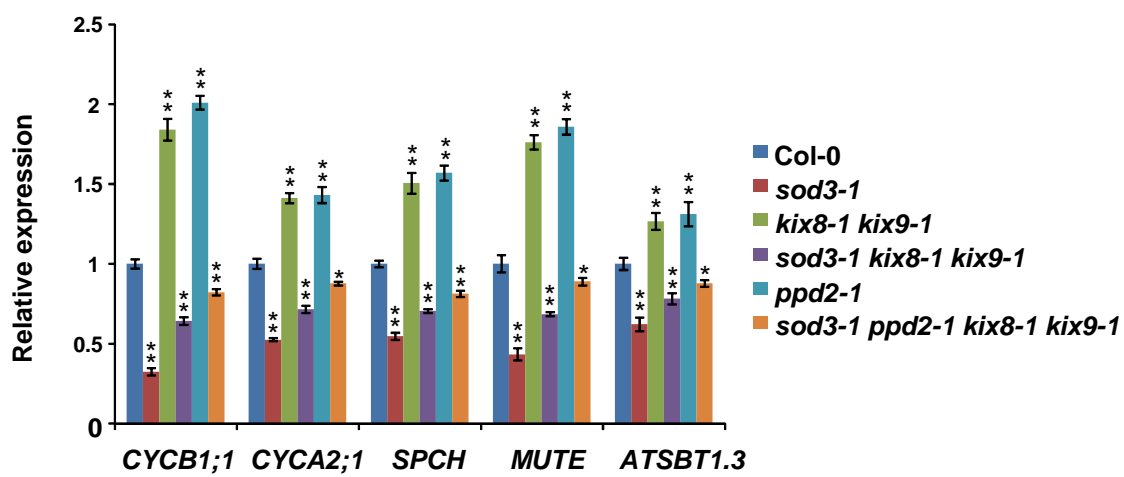

Figure S7

Supplement: S7 Fig — *P<0.05, **P<0.01 compared with the wild type (Student’s t-test). (PDF) [file pgen.1007218.s007.pdf]
