# Supplementary material for: STERILE APETALA modulates the stability of a repressor protein complex to control organ size in Arabidopsis thaliana
Source: PLoS Genet. 2018 Feb 5;14(2):e1007218. doi: 10.1371/journal.pgen.1007218 (PMC5814100; doi:10.1371/journal.pgen.1007218)
Supplement: S1 Table — (PDF) [file pgen.1007218.s008.pdf]

Supplemental Table 1. List of primers used in this study

Primer Name	Sequence
KIX8-F/R-myc	GAAGAGGACTTGAATTCGGTACCCATGCCGAGGCCAGGACCAAGACCTT
KIX8-F/R-myc	TAGGCTACGTAGGATCCACTAAAGGAAGTCTCCACACAAAGTT
KIX9-F/R-myc	GAAGAGGACTTGAATTCGGTACCCATGCCGAGGCCAGGGCCAAGACCGT
KIX9-F/R-myc	TAGGCTACGTAGGATCCATCAGTTGTTATTGTTGCTGCTTATC
TPL-F/R-myc	GAGGACTTGAATTCGGTACCCATGTCTTCTCTTAGTAGAGAGC
TPL-F/R-myc	CGATTTCGAACCCGGGGTACCTCATCTCTGAGGCTGATCAG
SAP-RT-F	GAATCTCTCGCCGTTCAAGT
SAP-RT-R	CCCAAACTTGGCGAGATAGT
ACTIN2-F	GAAATCACAGCACTTGCACC
ACTIN2-R	AAGCCTTTGATCTTGAGAGC
CYCD3;2-RT-F	GTTGGAGCACAATCCAAGC
CYCD3;2-RT-R	AGTGGAGACATTCCAGGAGC
CYCD3;3-RT-F	GATTTTCAAGTGGAAGAAGCAAG
CYCD3;3-RT-R	CGAAAAACGAGATTGGAGTC
CYCDB1;1-RT-F	ATTATGTCTTCCTAGCTCGTT
CYCDB1;1-RT-R	TGCATTACGCCTAACTCAGC
CYCA2;1-RT-F	ACCGAGTAATAAACGAATGGC
CYCA2;2-RT-R	GTTTCTTTACCACCTCGCTTG
SPCH-RT-F	ATAATAAGCAAGATGGGCAAC
SPCH-RT-R	TTTGACGTAGAAACAAGGCAT
MUTE-RT-F	CGAGCATCTTAAATCCCTTCGT
MUTE-RT-R	TCTTTCGACGTTTCTTGGACT
TSBT1.3-RT-F	AATATTCTTGCAGCTTGGAC
TSBT1.3-RT-R	GATCAAAGCAGCTACACCAC
PPD1-RT-F	AAAGACAGAAAATTCTCAAAGG
PPD1-RT-R	AGTGGAGATCCGGTGCAGCC
PPD2-RT-F	ATTTTCTAAGACCAAGAAGGC
PPD2-RT-R	CGCAATGCCCTGTGCCACT
KIX8-F/R-AD	GCCATGGAGGCCAGTGAATTCATGCCGAGGCCAGGACCAAGAC
KIX8-F/R-AD	CAGCTCGAGCTCGATGGATCCCTAAAGGAAGTCTCCACACAAAG
KIX9-F/R-AD	GCCATGGAGGCCAGTGAATTCATGCCGAGGCCAGGGCCAAG

	ACCGTACGAC
KIX9-F/R-AD	CAGCTCGAGCTCGATGGATCCTCAGTTGTTATTGTTGCTGCTT
PPD1-F/R-AD	GCCATGGAGGCCAGTGAATTCATGGATGTCGGAGTTTCACCG
PPD1-F/R-AD	CAGCTCGAGCTCGATGGATCCTTAAATGCCTTCACTGTTTAG
PPD2-F/R-AD	GCCATGGAGGCCAGTGAATTCATGGATGTAGGAGTTACTACGGC
PPD2-F/R-AD	CAGCTCGAGCTCGATGGATCCTTAATTATCTTCGCTGTTTAG
KIX9KIX-F/R-AD	GACGTACCAGATTACGCTCATATGATGCCGAGGCCAGGGCCAAGAC
KIX9KIX-F/R-AD	TTCACTGGCCTCCATGGCCATATGTTAGGCTTTGGAATACATGATTTCTT
KIX9C-F/R-AD	GACGTACCAGATTACGCTCATATGGACTCAACTAACTTGGACAACAT
KIX9C-F/R-AD	TTCACTGGCCTCCATGGCCATATGTTAGTTGTTATTGTTGCTGCTTATC
KIX9KIX-B-F/R-AD	GACGTACCAGATTACGCTCATATGATGCCGAGGCCAGGGCCAAGAC
KIX9KIX-B-F/R-AD	TTCACTGGCCTCCATGGCCATATGTTATTGGCTAAGATAACATCTCGGGT
KIX9B-C-F/R-AD	GACGTACCAGATTACGCTCATATGAATTCTGAGGCTGAGTATATG
KIX9B-C-F/R-AD	TTCACTGGCCTCCATGGCCATATGTTAGTTGTTATTGTTGCTGCTTATC
KIX9KIX-C-F-ADM	AAGAAATCATGTATTCCAAAGCCGACTCAACTAACTTGGACAACAT
KIX9KIX-C-R-ADM	ATGTTGTCCAAGTTAGTTGAGTCGGCTTTGGAATACATGATTTCTT
KIX8KIX-B-F/R-AD	GACGTACCAGATTACGCTCATATGATGCCGAGGCCAGGACCAAGAC
KIX8KIX-B-F/R-AD	TTCACTGGCCTCCATGGCCATATGTTAGAGGTAAGTCCTTCCGCTACT
KIX8B-C-F/R-AD	GACGTACCAGATTACGCTCATATGAATTCCGAGGAAGAGTATACAG
KIX8B-C-F/R-AD	TTCACTGGCCTCCATGGCCATATGTTAAAGGAAGTCTCCACACAAAGT
KIX8KIX-F/R-AD	GACGTACCAGATTACGCTCATATGATGCCGAGGCCAGGACCAAGAC
KIX8KIX-F/R-AD	TTCACTGGCCTCCATGGCCATATGTTAGGCTTTAGAGTACATGATTTC
KIX8C-F/R-AD	GACGTACCAGATTACGCTCATATGGGTCCTAAAATTCAGGAACCGGT
KIX8C-F/R-AD	TTCACTGGCCTCCATGGCCATATGTTAAAGGAAGTCTCCACACAAAGT

KIX8KIX-C-F-ADM	GGTCCTAAAATTCAGGAACCGGTGAAATCATGTACTCTAAAGCC
KIX8KIX-C-R-ADM	GGCTTTAGAGTACATGATTTCACCGGTTCCTGAATTTTAGGACC
KIX8-F/R-HIS	ATGGGTCGCGGATCCGAATTCATGCCGAGGCCAGGACCAAGAC
KIX8-F/R-HIS	TGCGGCCGCAAGCTTGTCGACCTAAAGGAAGTCTCCACACAAAG
KIX9-F/R-HIS	ATGGGTCGCGGATCCGAATTCATGCCGAGGCCAGGGCCAAGAC
KIX9-F/R-HIS	TGCGGCCGCAAGCTTGTCGACTCAGTTGTTATTGTTGCTGCTT
KIX8-F/R-LUC	GAGCTCGGTACCCGGGGATCCATGCCGAGGCCAGGACCAAGACCTT
KIX8-F/R-LUC	GCGTACGAGATCTGGTCGACAAGGAAGTCTCCACACAAAGTT
KIX9-F/R-LUC	GAGCTCGGTACCCGGGGATCCATGCCGAGGCCAGGGCCAAGACCGT
KIX9-F/R-LUC	GCGTACGAGATCTGGTCGACGTTGTTATTGTTGCTGCTTATC
SAP-F/R-LUC	GAGCTCGGTACCCGGGGATCCATGTCTACCTCCTCCTCTTCTT
SAP-F/R-LUC	GCGTACGAGATCTGGTCGACCAGTGCACCGAAATCCCATAG
